# Identification of Key Proteins Related to Cashmere Fiber Diameter by Integrated Proteomics and Bioinformatic Analyses in the Alpas and Alxa Goat Breeds

**DOI:** 10.3390/genes15091154

**Published:** 2024-09-01

**Authors:** Chongyan Zhang, Qing Qin, Yichuan Wang, Zhixin Wang, Zhihong Liu

**Affiliations:** 1 Animal Science Department, Inner Mongolia Agricultural University, Hohhot 010018, China; zhangcy9753@163.com (C.Z.);; 2 Inner Mongolia Key Laboratory of Sheep & Goat Genetics Breeding and Reproduction, Hohhot 010018, China; 3 Key Laboratory of Mutton Sheep & Goat Genetics and Breeding, Ministry of Agriculture and Rural Affairs, Hohhot 010018, China

**Keywords:** goat, keratin, proteomics

## Abstract

Background: Goats (*Capra hircus*) have always been a source of fiber for human use and hold an important place in international high-end textiles. Fiber diameter is the most concerning economic indicator for producers. Understanding the formation mechanism of fiber diameter and its related key proteins can help optimize and control the production of cashmere. Methods: Cashmere goats (*n* = 36) of the Alpas (*n* = 18) and Alxa (*n* = 18) breeds, with a similar age (2 years old) and live weight (25–26 kg), were selected from the Yiwei White Cashmere Goat Breeding Farm, Erdos, Inner Mongolia. Using phenotypic indicators, we evaluated the diameter of the cashmere fibers in Alxa and Alpas goats. We also used electron microscopy to examine the cashmere fiber’s structure and label-free liquid chromatography–tandem mass spectrometry to determine the protein content of the two cashmere fibers. The proteins affecting fiber diameter were identified and analyzed by Western blot, Co-Immunoprecipitation, and bioinformatics analysis. Results: The average diameter of the Alxa breed was smaller (*p* < 0.05) than that of the Alpas breed (Alxa’s cashmere vs. Alpas’ cashmere). Proteomics technology enabled the highly confident detection of 171 proteins. A total of 68 differentially expressed proteins were identified in the two types of cashmere; 131 proteins were specifically expressed in Alpas goats, and 40 proteins were specifically expressed in Alxa goats. A key protein group that could cause variations in fiber diameter was found using the protein–protein interaction network. To ascertain the reason for the variation in fiber diameter, a structural study of the major protein groups was carried out. Conclusions: *KRT10*, *KRT14*, *KRT17*, and *KRT82* are the main proteins impacting the diameter difference, and they have a substantial effect on the average fiber diameter.

## 1. Introduction

Animal-derived fibers are widely used in many industries and traditional products, and they are important commodities in the global textile industry. The performance and commercial value of different animal fiber varieties vary greatly. For example, mohair or cashmere from goat (*Capra hircus*) [1] and alpaca (Lama pacos) [2]; vicuña wool (Vicugna) [3]; and fibers from other less familiar species such as qiviut from musk ox (Ovibos moschatus) are internationally recognized, particularly in the segment of traditional craft products. However, cashmere fiber is still the most valuable and extensively utilized animal fiber. It is produced throughout the world and assumes a strategic role in the economies of several nations, such as Australia, New Zealand, Morocco, Iran, Pakistan, etc. Cashmere fiber is only obtained from goats and is used in luxury clothing and traditional crafts.

As the largest cashmere producer and exporter, China’s cashmere output accounts for 50% of the world’s cashmere production, of which, Inner Mongolia’s cashmere fiber output accounts for about 30% [4]. Alpas and Alxa goats, which are used in Inner Mongolia to obtain cashmere, are distributed in different regions. There is a typical heterogeneity of coat types, with wool being produced by primary hair follicles and cashmere being produced by secondary hair follicles [5]. Goats are vital for the daily lives of some human societies, and they are famous for their excellent fiber production characteristics; as an important textile, cashmere has high economic value and is known as soft gold [5,6,7].

Cashmere is a kind of protein fiber, which is composed of keratin (*KRT)* and keratin-associated proteins (*KRTAPs*) [8]. The former assembles into intermediate filaments (IFs), and the latter cross-links the keratins in the IFs to form a semi-rigid matrix; they can regulate the mechanical stress of cells in the skin, thus having an impact on the elastic properties of the skin [9,10]. It is thought that these two types of proteins play an important role in controlling the properties of fibers [11]. *KRT* and *KRTAPs* are the main structural proteins in cashmere and sheath, and their contents are of great significance to the fiber quality and hair morphology of goats [12,13]. Cashmere is composed of two parts, the cuticle and the cortex. The cortex is mainly responsible for the mechanical properties of the fiber such as its strength and rigidity [14,15], and the cuticle plays a crucial role in the durability, feel, and contractility of the fiber [16]. Yu identified transcripts of *KRT40*, *KRT82*, and *KRT84* that are only expressed in the fiber cuticle by cDNA sequences, while *KRT32*, *KRT35*, and *KRT85* were present in the cuticle and fiber cortex [17]. Therefore, the keratin family is a key family that can be used to understand the structure, character, and function of fibers.

Cashmere fiber diameter (CFD) is one of the fiber traits with high economic benefits, and the identification of proteins that regulate this trait will provide an opportunity to increase productivity and improve product quality and diversity [18,19]. In this study, we used liquid chromatography–tandem mass spectrometry to prove the relationship between cashmere fiber fineness and the fiber proteome of different breeds of goats. The results obtained may provide a physiological and commercial insight into the effects of protein on the characteristics of different breeds of cashmere-producing goats.

## 2. Materials and Methods

### 2.1. Experimental Animals

The permanent position of cashmere goats was 107°928814 east longitude, 39°208408 north latitude, 1800 m above sea level, and 678.8% yearly sunlight. The goats were in good health and were not pregnant or lactating. It was ensured that the goats had enough energy, protein, minerals, and vitamins each day; their living quarters were dry and airy, and the right temperature and humidity were provided.

Female cashmere goats (*n* = 36) from the Yiwei White Cashmere Goat Breeding Farm, Erdos, Inner Mongolia, of a similar age and belonging to the breeds Alpas (*n* = 18) and Alxa (*n* = 18) were used to collect the cashmere samples for further analyses. The test samples (cashmere fiber) were cut from the position of the scapula of the goat and were stored at ambient temperature for subsequent experiments where they were processed for protein extraction. Samples were collected according to the Guidelines for Experimental Animals of the Ministry of Science and Technology (Beijing, China), and they were approved by the Scientific Research and Academic Ethics Committee of Inner Mongolia Agricultural University and the Biomedical Research Ethics of Inner Mongolia Agricultural University (Approval No. [2020] 056). All samples were in accordance with the international guidelines for animal research.

### 2.2. Measurement of Important Economic Characteristics

The natural length, diameter, and strength of the Alxa and Alpas fibers were measured in the laboratory; samples were washed, dried, and tested under conditions of constant temperature and humidity (20 ± 2 °C, 65 ± 4%). The entire tuft of raw hair was extracted at its natural length, and care was taken to preserve the original shape of the hair bundle when measuring the Alxa and Alpas samples independently. The natural length of the fiber samples was measured using a steel ruler under normal humidity and temperature settings. The length was measured according to the IWTO 17-1985 standard (Determination of Fiber Length Distribution Parameters using the Almeter). Cashmere fiber was cut into short segments, evenly scattered on the slide, on the machine for testing. The diameter was measured using an OFDA 2000 (BSC Electronics, Perth, Australia) optical fiber diameter analyzer according to the IWTO47-2013 standard (Method of Determining Fiber Diameter Distribution Parameters and Percentage of Medullated Fibers in Wool and Other Animal Fibers by the Projection Microscope). The cashmere fiber was stretched naturally and tested on the machine. Fiber strength was measured with a YG006 Electronic Single Yarn Strength Tester (Bohui Instrument, Xi’an, China) according to the IWTO-30-2007 standard (Determination of Staple Length and Staple Strength). An EVO10 scanning electron microscope (Carl Zeiss, Oberkochen, Germany) was used to observe the structure of cashmere fiber. EXCEL was used for data processing, SPSS 25.0 was used for One-Way Analysis of Variance, and *p* < 0.05 indicated a significant difference.

### 2.3. Protein Extraction and Digestion

Water was used to rinse the 0.5 g Alpas and 0.5 g Alxa samples. Each sample was then given 1 mL of a dichloro-methane and methanol combination, and the samples were shaken for two hours at 50 °C. The samples were centrifuged and then dried at 50 °C. The samples were placed into a pyrolysis buffer containing protease inhibitor (Roche, Basel, Switzerland) and 1% sodium dodecyl sulfate (Kulabor, Shanghai, China) and then placed in an oven at 37 °C for 48 h. The samples were then removed from the oven and centrifuged at 1000× *g* at 4 °C for 20 min. The supernatants were collected for further study, and the protein concentrations were measured with a bicinchoninic acid (BCA) kit (Tiangen, Shanghai, China).

**Figure 1:**
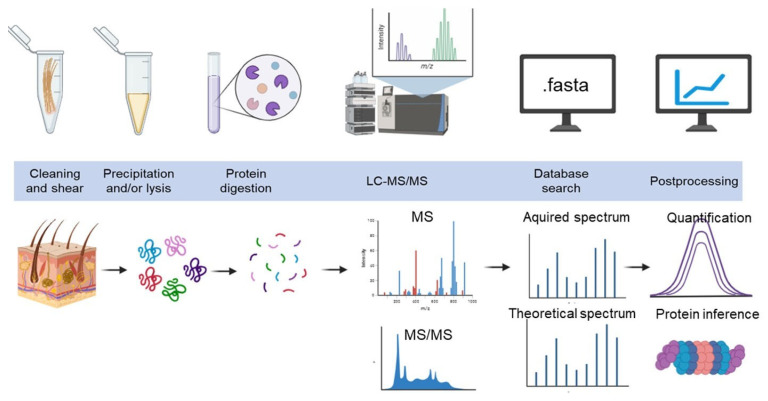
Generic workflow for proteomic sample preparation, data acquisition, and analysis. Proteins are extracted from whole cashmere samples or enriched subfractions using optimized homogenization and protein extraction methods. Proteins are digested into peptides and measured on an LC-MS/MS system. Peptides are identified from MS/MS spectra by database matching and quantified based on the peak areas of intact peptide (survey MS spectra) or fragment ion signals (tandem MS spectra) provided by the mass spectrometer along the chromatographic time scale.

The procedure was performed basically according to Xie et al. [20]. A total of 200 µL of 8 M urea and 10 mM dldithiothreitol were added to the protein solution samples (see above), which contained 100 µg of protein. The samples were then incubated for one hour at 37 °C. After centrifugation at 12,000× *g* for 40 min, the precipitate was retained, and the supernatant was discarded. After that, the precipitate was mixed with 200 µL of 8 M urea and stirred. The tubes were centrifuged twice at 12,000× *g* for 30 min, and after each centrifugation, the supernatant was discarded. Subsequently, 200 µL of 50 mM iodoacetamide was introduced into every tube, left to stand in the dark for half an hour, centrifuged for ten minutes at 12,000× g, and the supernatant was disposed of. Then, 100 μL of 100 mM ammonium bicarbonate was added to each tube, and the samples were centrifuged at 12,000× *g* for 20 min. This step was performed three times, and after each centrifugation, the supernatant was discarded. The samples were incubated overnight with 4.3 μL (2.15 μg) trypsin (Promega, Madison, WI, USA) at 37 °C and centrifuged at 12,000× *g* for 30 min. The supernatant was collected, and the precipitate was discarded. Then, 50 µL of 100 mM ammonium bicarbonate was added to each tube. The samples were centrifuged at 12,000× *g* for 30 min, the supernatant was collected, and this step was repeated. The supernatant was freeze-dried and stored at −20 °C (Figure 1).

Protein identity and quantity are then inferred based on peptide sequence assignments. Abbreviations: LC-MS/MS, liquid chromatography coupled with tandem mass spectrometry; MS, mass spectrometry; MS/MS, tandem mass spectrometry; *m*/*z*, mass-to-charge ratio.

### 2.4. Label-Free LC/MS Quantitative and Qualitative and Profiling

Protein Pilot v4.5 software (Sciex, Framingham, MA, USA) and the UniProt/SWISS-PROT/Capra hircus database (https://www.uniprot.org/# accessed on 1 June 2024) were used for peptide identification. The results were filtered with a 1% false discovery rate (FDR). The selected search parameters included the use of trypsin as the enzyme, allowing up to two missed cleavage sites. The peptide mass tolerance was 15 ppm, and the fragment mass tolerance was 20 mmu. The data were loaded into PeakView v2.1 (Sciex, Framingham, MA, USA) software, and the ion library generated by Protein Pilot was used to search the Sequential Windowed Acquisition of all Theoretical fragmentions (SWATH) database. PeakView processed the target and nontarget data to generate the extracted ion chromatograms (XICs). Then, MarkerView v3.0 software (Sciex, Framingham, MA, USA) was used to explain and quantitatively analyze the results. MarkerView allows for a rapid review of data to determine the differentially expressed proteins (DEPs). Principal component analysis (PCA) and volcano plot analysis, which combined the fold change analysis and *t*-tests, were performed. A fold change >2 or fold change <0.5 and statistical significance (*p*-value < 0.05) were used to identify DEPs.

### 2.5. Bioinformatics Analysis

GO functional annotation was performed using the david database (https://david.ncifcrf.gov/, accessed on 1 June 2024) with default parameters and Capra hircus genome annotation as the background. The results were obtained using methods including Biological Process (BP), Molecular Function (MF), and Cellular Component (CC) analysis [21].

Lipoprotein–protein interaction analysis of the differentially expressed proteins between goats was carried out using the Search Tool for the Retrieval of Interacting Genes/Proteins (STRING: https://cn.string-db.org/, accessed on 1 June 2024) database [22], as already described [23]. The network was constructed with a minimum required interaction score of >0.4. Cytoscape v.3.9.0 software (Cytoscape Consortium, San Diego, CA, USA) was used to visualize the network [24,25]. In addition, the Cytoscape add-on CytoHubba was used to explore the hub proteins of the PPI network using the McCreight (MCC) method [26].

The predict protein database system (https://predictprotein.org/, accessed on 1 June 2024) was used for the analysis of the secondary structure of key proteins identified in both breeds [27]. SWISS-MODEL (https://swissmodel.expasy.org/, accessed on 1 June 2024) is a server for automated comparative modeling of three-dimensional (3D) protein structures. It was used to predict the tertiary structure of key proteins identified in both breeds [28,29,30].

### 2.6. Western Blotting

The source proteins obtained above were boiled at 100 °C for 3 min to become denatured proteins. The procedure was referenced according to Xie et al. [20]. The denatured proteins were separated using SDS-PAGE and transferred to PVDF membranes (Bio-Rad, Lincoln, NE, USA). PVDF membranes were incubated overnight in a dilution of mouse monoclonal anti-rabbit *KRT14* primary antibody (Abcam, Cambridge, MA, USA; diluted 1:1000). The membranes were incubated with fluorescent goat anti-mouse secondary antibody (LI-COR Biosciences, Lincoln, NE, USA; dilution 1:30,000) for 1 h after washing three times. The results were finally observed with a LI-COR Odyssey 10 NIR imager (LI-COR Biosciences). The same denatured proteins (as above) were separated using SDS-PAGE and transferred to PVDF membranes (Bio-Rad). Actin beta expression was similar in the Alxa and Alpas breeds, so it was chosen as a reference protein. PVDF membranes were incubated overnight in a dilution of mouse monoclonal anti-rabbit actin primary antibody (Abcam; diluted 1:1000). The membranes were incubated with fluorescent goat anti-mouse secondary antibody (LI-COR Biosciences; dilution 1:30,000) for 1 h after washing three times. The results were finally observed with a LI-COR Odyssey 10 NIR imager (LI-COR Biosciences). The PVDF membranes were cut before hybridization with antibodies. The band size of *KRT14* is 52 kD, and before hybridization with antibodies, we only retained the 40–70 kD portion. The band size of actin is 45 kD, and we only retained a 30–60 kD portion before hybridization with an antibody.

### 2.7. Co-Immunoprecipitation (Co-IP)

The source proteins obtained above were incubated with corresponding antibodies (*KRT14*) and Protein A/G Magnetic Beads (MCE) at 4 °C overnight. Subsequently, the samples were separated by a magnetic separator denatured by an SDS loading buffer and examined by mass spectrum analysis.

## 3. Results

### 3.1. Performance of Fiber Quality Traits

**Figure 2:**
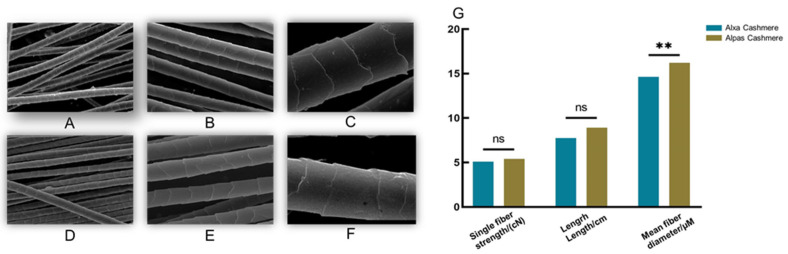
Cashmere economic traits. (**A**) Alpas-type cashmere scale structure (300×). (**B**) Alpas-type cashmere fiber scale structure (1000×). (**C**) Alpas-type fiber scale structure (2000×). (**D**) Alxa-type fiber scale structure (300×). (**E**) Alxa-type cashmere scale structure (1000×). (**F**) Alxa-type cashmere scale structure (2000×). (**G**) Two types of fiber phenotypic traits (ANOVA: ** *p* < 0.01; ns: not statistically significant).

The physical and chemical differences between the fibers are determined by the differences in their scale structures. The Alxa fibers are primarily rings, whereas the Alpas fibers are primarily oblique rings (Figure 2A–F). The average fiber diameter is the most important cashmere index for producers. As the fiber diameter becomes smaller, the income rises. The measurement results show that producers will have no other option when it comes to breeding Alpas cashmere goats in the future. The firmness and elasticity of textiles are determined by the elongation and fracture resistance of fibers, which are measured by fiber strength, an essential quality index. From the results, it can be concluded that Alpas’ cashmere has stronger tensile resistance and better quality after processing. (Figure 2G).

### 3.2. Proteome Analysis during Fiber Development

**Figure 3:**
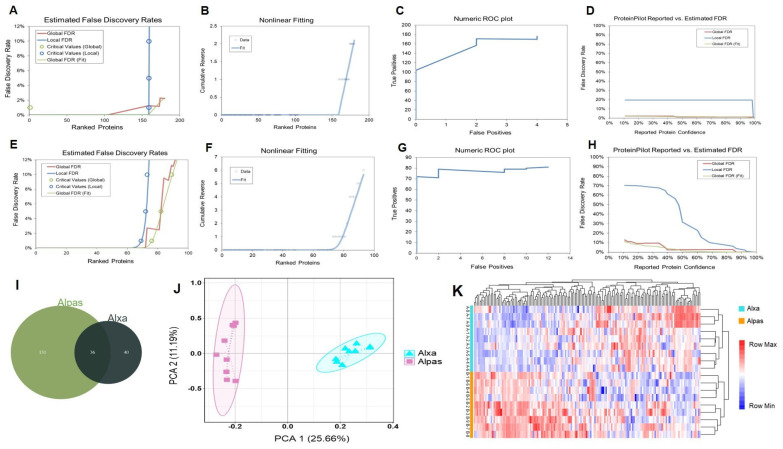
LC-MS/MS analysis and identification of the protein. (**A**) Estimated false discovery rates of Alxa fiber protein. (**B**) Nonlinear fitting of Alxa fiber protein. (**C**) Numeric ROC plot of Alxa fiber protein. (**D**) ProteinPilot reported vs. estimated FDR of Alxa fiber protein. (**E**) Estimated false discovery rates of Alpas fiber protein. (**F**) Nonlinear fitting of Alpas fiber protein. (**G**) Numeric ROC plot of Alpas’ fiber protein. (**H**) ProteinPilot reported vs. estimated FDR of Alpas fiber protein. (**I**) Venn diagram showing the distribution of proteins in the different cashmere types. (**J**) Two-dimensional scatter plot of quantitative principal component analysis of protein among samples. (**K**) The heatmap depicts relative abundance levels (log 10) of all proteins quantified from SWATH data; colors represent differences in the abundance of proteins in the column; two major sample clusters (rows) align with the Alpas and Alxa area; and genotypes show some propensity to cluster within these two major groupings.

We studied proteome variations between Alxa and Alpas’ cashmere, which represent the important nodes of the dynamic change in fiber diameter, in order to investigate the key proteins and molecular mechanisms of fiber development that govern fiber qualities. Unmarked mass spectrometry was used to study the white matter based on the UniProt/SwissProt/Capra hircus database, with a false discovery rate (FDR) of ≤0.01. In total, 76 proteins and 800 peptides were identified in Alxa (Figure 3A–D), and 167 proteins and 3079 peptides were identified in Alpas (Figure 3E–H). As predicted, Venn analysis also revealed that whilst some proteins were expressed primarily in Alxa, others were expressed more widely. (Figure 3I). A total of 208 proteins were found to be expressed in all cashmere types, Additionally, there were 40 proteins only expressed in Alxa and 131 proteins only expressed in Alpas.

An initial assessment of the data was performed using unsupervised PCA, revealing that samples are stratified, where PC1 (location region) and PC2 (protein expression) explained 36.85% of the variation in the dataset (Figure 3J). Samples from each cashmere cluster together, indicating that the effect of each variety outweighs the effect of the line. Hierarchical clustering analysis (HCA) showed that the sample source is divided into two areas, and the results and collecting the data of the actual sample are the same (Figure 3K). This further supports the effect of the variety on the proteome composition across the two cashmere samples as well as highlights the substantial shift in the proteins’ abundances between the locations.

### 3.3. Proteome Analysis of Differentially Expressed Proteins during Fiber Development

**Figure 4:**
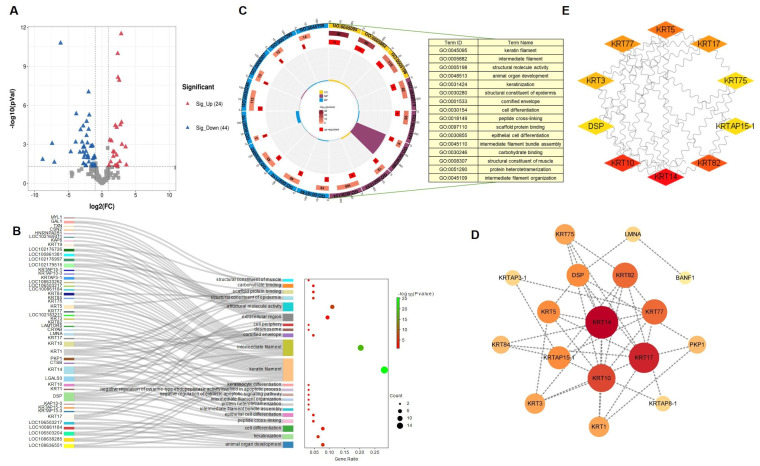
Differentially expressed protein analysis. (**A**) Volcano map of differentially expressed proteins. The abscissa represents the difference multiple of the differential protein (log2 value), and the vertical axis represents the *p*-value (−log10 value). Gray represents the protein with no significant difference, red represents the upregulated protein (FC > 2, *p*-value < 0.05), and blue represents the downregulated protein (FC < 0.5, *p*-value < 0.05). (**B**) GO function analysis of differentially expressed proteins based on the DAVID database. (**C**) GO function analysis of differentially expressed proteins based on the DAVID database at a *p*-value of <0.05. (**D**) Protein-protein interaction regulatory network. DEPs identified in the Alpas and Alxa groups to construct a regulatory network using STRING (https://cn.string-db.org/ accessed on 18 August 2024) to visualize the interaction with evidence such as network edge importance. The active interaction sources were Text Extraction, Experiments, Database, Coexpression, Neighborhood, Gene Fusion, and Co-occurrence, with a required minimum interaction score of medium confidence (0.4). (**E**) The top 10 proteins with the highest degree of PPI network connectivity were identified by the MMC method using CytoHubba.

The DEPs were then identified to further reveal the molecular characteristics of cashmere with two diameters. Volcano plots were used to showcase proteins that showed fold changes of ≥2 or <0.05 and a *p*-value of < 0.05 for each set of comparisons (Figure 4A). In groups Alxa and Alpas, 24 proteins were more abundant and 44 showed reduced abundance. The GO enrichment analysis of DEPs revealed the protein functions of different regions induced by DEPs (Figure 4B). Keratin filament, intermediate filament, and structural molecule activity were the most enriched GO terms in Alxa and Alpas cashmere under two different geographical areas. Notably, most of the DEPs involved in these functions are keratins and keratin-associated proteins. Based on the condition of *p* < 0.05, GO analysis is mainly enriched in keratin filament and intermediate filament terms. The sixteen pathways enriched are shown in Figure 4C; among them, *KRT14*, *KRT17*, and *KRT10* not only play a role in keratin filament and intermediate filament terms but also participate in 14 other pathways, which may be key pathways to regulate the phenotypic character of cashmere. To determine the interaction relationship between the proteins expressed by these DEPs in the cashmere growth mechanism, the PPI network was constructed using STRING (https://cn.string-db.org/, accessed on 1 June 2024) (Figure 4D). Among the 68 DEPs, the STRING analysis revealed that 17 proteins interacted with each other. In addition, three of the top proteins with relatively high connectivity degrees (>6) were *KRT14* (degree = 9), *KRT17* (degree = 8), and *KRT10* (degree = 7). Furthermore, based on the analysis with Cytoscape McCreight, the top 10 proteins in the network were acquired (Figure 4E). Among the top ten in the network by the MCC method, the top three proteins were *KRT14* (score = 23), *KRT10* (score = 20), and *KRT82* (score = 16).

### 3.4. Key Protein Structure Domain Analysis

Proteins are essential to almost every biological activity in living things. They account for 50% of the dry mass of cells and play a role in everything the organism does. They are known as the most structurally complex biological molecules. Nonetheless, they are all composed of the same 20 amino acids. Large polypeptide chains can be formed by forming peptide bonds between amino and carboxyl groups on two different amino acids.

**Figure 5:**
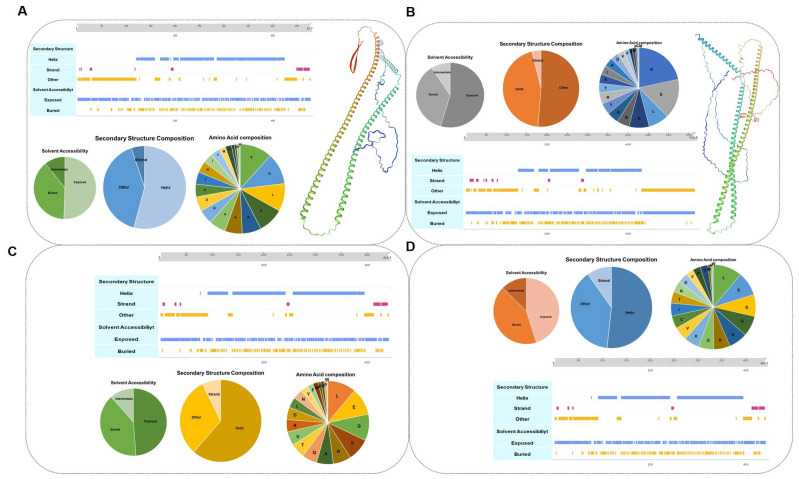
Structural analysis of key proteins. (**A**) Secondary structure analysis and tertiary structure prediction of keratin 14. (**B**) Secondary structure analysis and tertiary structure prediction of keratin 10. (**C**) Secondary structure analysis and tertiary structure prediction of keratin 17. (**D**) Secondary structure analysis and tertiary structure prediction of keratin 82.

The *KRT14* sequence length of 477 encodes a total of 75 amino acids. Predict protein server prediction results show that the highest proportion of amino acid composition is serine (S). In this protein, 54% is Helix, 5% is Strand, and 41% is a nonhelical and non-folded random structure. On Solvent Accessibility, 39% of the area is Buried and 51% is Exposed (Figure 5A). The *KRT10* sequence length of 564 encodes a total of 75 amino acids. The highest proportion of amino acid composition is glycine (G). In this protein, 45% is Helix, 4% is Strand, and 51% is a nonhelical and non-folded random structure. On Solvent Accessibility, 36% of the area is Buried and 55% is Exposed (Figure 5B). The *KRT17* sequence length of 441 encodes a total of 75 amino acids. The highest proportion of amino acid composition is leucine (L). In this protein, 62% is Helix, 7% is Strand, and 31% is a nonhelical and non-folded random structure. On Solvent Accessibility, 40% of the area is Buried and 49% is Exposed (Figure 5C). The *KRT82* sequence length of 514 encodes a total of 45 amino acids. The highest proportion of amino acid composition is L. In this protein, 52% is Helix, 10% is Strand, and 38% is a nonhelical and non-folded random structure. On Solvent Accessibility, 42% of the area is Buried and 45% is Exposed (Figure 5D).

Among the four keratins, the top four amino acids are S, G, L, and glutamic acid (E). S is a polar, uncharged amino acid acting on backbone/side chain molecules of the amino acid and can be used to form hydrogen bonds; G and L as nonpolar, aliphatic amino acids acting on backbone molecules of the amino acid are used to form hydrogen bonds; glycine can cause a bend when used in an alpha helix chain (secondary structure); and E as an acidic amino acid can be used to form hydrogen bonds (backbone/side chain molecules) and salt bridges (side chain molecules only). Hydrogen bonds can help protein molecules maintain a stable three-dimensional structure. Interactions between protein molecules are important for many biological processes inside cells, and hydrogen bonds also help protein molecules interact. Thus, it can be inferred that these four proteins have strong stability and interact with each other to affect fiber diameter.

### 3.5. Western Blot Analysis of KRT14 Expression

**Figure 6:**
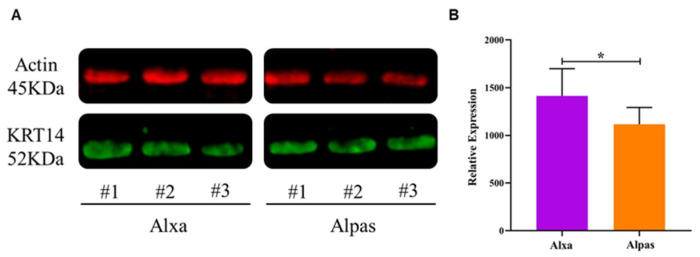
Western blot profiles of *KRT14*. (**A**) Three biological replicates from the cashmere fiber of Alxa and Alpas. (**B**) Relative intensity of protein is expressed as the mean ± standard deviation. The asterisk represents levels of significance (*t*-test: * *p* < 0.05).

Among all the DEPs, *KRT14* was strongly correlated with other *KRTs* in PPI network analysis. Thus, the *KRT14* protein was screened by Western blot analysis (Figure 6A). The expression of *KRT14* in Alxa was higher than that in Alpas (*p* < 0.05) (Figure 5B). This pattern was consistent with the quantitative proteomics results, further indicating the accuracy of the proteomics results.

### 3.6. KRT14 Interacts with Other Proteins

We used *KRT14* to conduct the Co-IP analysis in order to obtain further insight into the mechanisms of keratin interaction in fiber. By mass spectrometry analysis of immunoprecipitated proteins, 39 potential *KRT14*-binding proteins were identified compared to the control group (Table 1). Of these, LOC108633262, keratin 17, myosin heavy chain 2, and LDL receptor-related protein 4 play an important role in the composition of fibers. Compared with the interacting proteins predicted by the STRING database, the IP results identified more proteins targeted by *KRT14*, which affected each other as a protein group in the fiber and played a role together and also confirmed the strong interaction between *KRT17* and *KRT14*. At the same time, this result enriches the STRING database and is of great significance.

## 4. Discussion

In order to find and clarify relationships between the proteome and fiber diameter, this work examines the proteome phenotypes of two breeds of cashmere goats. The article assesses the influence of genetics on proteome phenotypes, pinpoints specific proteins that show an established association with fiber diameter, and clarifies the causes of the fineness differences between Alpas and Alxa cashmere fibers. This provided the groundwork for further improvements in cashmere fiber fineness and a rise in revenue.

Previous comparative studies on cashmere were mostly based on genomic and transcriptomic methods [31,32,33]. However, as posttranscriptional mechanisms dominate the regulation of gene expression, proteomic investigations in cashmere are crucial. In the Alpas and Alxa cashmere fibers, our label-free proteomics revealed a significant number of structural molecular activities and keratin filament processes, the majority of which are structural proteins, such as keratin and keratin-associated proteins. Keratin is a nonnutritive protein with fiber properties, and it is widely found in the epidermis of humans and animals. As the main component of fiber, it determines the structural characteristics of the fiber [34,35]. Among the proteins that were identified, some proteins (e.g., Keratin38, Keratin5, Keratin14, Keratin25, Keratin71, Keratin31, Keratin36, and Keratin85) were also highlighted in other investigations on the skin of goat or other farm animals to modulate the phenotypic traits of the fibers [17,35,36,37]. The protein list found in this study is an improvement over previous research and could spur further research into the mechanisms underlying the differences in the average fiber diameter of cashmere, which could yield new insights into the species and its production. It also provides by far the broadest picture of the Inner Mongolia cashmere fiber proteome.

The researchers used RNA sequencing to compare gene expression in skin samples from cows with different hair diameters and found that *KRT14* was expressed at higher levels in the skin with thicker hair diameters and at lower levels in the skin with finer hair diameters, indicating that *KRT14* is involved in regulating the formation process of hair thickness [38]. In this study, *KRT14,* as an upregulated protein, had high expression in the thicker fiber of Alpas and low expression in the finer fiber of Alxa. *KRT14* participated in the process of epithelial cell differentiation and protein hetero-tetramerization, involving keratin filament and intermediate filament organization; it also plays a role in structural molecule activity, and it is speculated that *KRT14* for cashmere fineness has a certain regulation effect. When the *KRT17* gene was deleted in mice, the mice’s hair fineness was considerably reduced. The effects of loss of *KRT17* on hair development and fineness are similar to the symptoms of hormone-sensitive hair loss. These findings imply that *KRT17* may have some influence on the growth and fineness of hair [39]. The effect of *KRT17* on hair diameter is also supported by several studies in humans, specifically, the higher the level of *KRT17* expression, the larger the hair diameter [40,41]. In this study, *KRT17* expression was higher in Alpas’ thicker fibers than in Alxa’s finer fibers. Meanwhile, it is found that *KRT17* participates in the process of keratinization, involving keratin filament and intermediate filament organization; it also plays a role in structural molecule activity. It is speculated that *KRT17* plays a certain role in the regulation of fiber fineness. The expression level of *KRT10* in keratinocytes is related to the thickness of the stratum corneum and affects the synthesis of cortical proteins during hair follicle cell differentiation; overexpression of *KRT10* may cause excessive proliferation and differentiation of keratinocytes, resulting in larger keratinocytes in hair follicles, and thus coarser hair, further affecting hair growth and fineness [9,42,43]. In this study, it is found that *KRT10* participates in the process of peptide cross-linking and keratinocyte differentiation and plays a role in the structural constituent of the epidermis, and the expression level of *KRT10* in Alpas’ thicker fiber was higher than that in Alxa’s finer fiber; it is inferred that the fineness of cashmere fiber in goats is related to its content. *KRT82* may be involved in the process of hair growth and differentiation by regulating the synthesis and assembly of keratin, which affects hair diameter. The researchers used a technique to knock out the *KRT82* gene in the experimental sheep, and the results show that the yield of sheep’s wool was decreased as well as the length and diameter of wool [17,44,45]. Meanwhile, *KRT82* expression was higher in Alxa’s finer fibers than in Alpas’ thicker fibers, presumably because the regulation of fiber fineness may be influenced by the content of *KRT82*.

Studies have shown that estrogen has a major role in regulating hair development, growth, and loss. Estrogen can accelerate the growth and division of hair mother cells as well as the life cycle of hair follicles in order to increase the formation of hair. Furthermore, estrogen can influence glial, pigment, and stromal cell activity, which in turn can alter hair diameter. Specifically, estrogen can promote the division and proliferation of stromal cells, resulting in larger hair diameters; when estrogen levels decrease, stromal cell activity decreases, and hair diameter becomes thinner [46,47,48]. In the results of pathway analysis, *KRT14*, *KRT17*, and *KRT10* are involved in the estrogen signaling pathway, providing further evidence of their role in regulating fineness in cashmere fibers.

Organic sulfur-containing amino acids (SAAs), including cysteine and methionine, are the primary source of sulfur, a necessary mineral element for mammals [49]. The qualities of cashmere are directly impacted by the form, content, and structure of sulfur. Research has demonstrated that a higher sulfur concentration in fibers is linked to a bigger fiber diameter [49,50,51]. In this study, the sulfur content of *KRT82* (sulfur content = 6%), *KRT14* (sulfur content = 3%), *KRT10* (sulfur content = 2%), and *KRT17* (sulfur content = 3%) is highly expressed in Alpas. In the composition of amino acids, the expressions of *KRT14*, *KRT17*, *KRT10*, and *KRT82* were further demonstrated to affect cashmere fiber, especially on fineness.

A richer and more detailed explanation of the causes of the difference in the fineness of cashmere fibers at the proteomic level was provided. This study not only supports a comprehensive understanding of the mechanisms of cashmere fiber proteome but also provides a valuable reference for future related studies.

## 5. Conclusions

In Alpas and Alxa cashmere, *KRT10*, *KRT14*, *KRT17*, and *KRT82* play an important role in the average fiber diameter and are the key proteins affecting the diameter difference.

## Figures and Tables

**Table 1:** The interacting protein with KRT14 obtained through IP.

Number	Accession	Protein Name	Gene Name
1	A0A452EME4	Keratin 14	*KRT14*
2	A0A452FDX9	IF rod domain-containing protein	*LOC108633262*
3	A0A452FPA3	KIAA1217	*KIAA1217*
4	A0A452E4U1	Keratin 17	*KRT17*
5	A0A452FUI2	IF rod domain-containing protein	*KRT18*
6	A0A452G8R7	Myosin heavy chain 2	*MYH2*
7	A0A452EZ21	26S proteasome non-ATPase regulatory subunit 1	*PSMD1*
8	A0A452G5G4	Acid-sensing ion channel subunit 1	*ASIC1*
9	A0A452G188	Proline-rich 11	*PRR11*
10	A0A5D6VZD1	ABC-F family ATP-binding cassette domain-containing protein	*FZ040_10900*
11	A0A452G6J7	Obscurin, cytoskeletal calmodulin, and titin-interacting RhoGEF	*OBSCN*
12	A0A452FZK5	LDL receptor-related protein 4	*LRP4*
13	A0A452ERA9	Outer dynein arm-docking complex subunit 4	*ODAD4*
14	A0A5D6WAU5	Leukotoxin LktA family filamentous adhesin	*FZ040_03715*
15	A0A452E1K6	G protein-coupled receptor 68	*GPR68*
16	A0A5D6WDH4	DUF4405 domain-containing protein	*FZ040_00610*
17	A0A452FNB0	Glutamate-rich protein 1	*ERICH1*
18	A0A452ERJ7	OMA1 zinc metallopeptidase	*OMA1*
19	A0A452EE60	SHC adaptor protein 1	*SHC1*
20	A0A452E7K6	Serine/arginine repetitive matrix 2	*SRRM2*
21	A0A452FQE8	MLLT1 super elongation complex subunit	*MLLT1*
22	A0A452DL58	FH2 domain-containing protein	*N\A*
23	A0A452FQU4	Lengsin, lens protein with glutamine synthetase domain	*LGSN*
24	A0A452G661	Mucin like 3	*MUCL3*
25	A0A452FWB4	Corepressor interacting with RBPJ	*CIR1*
26	A0A452ECY9	Growth Differentiation Factor 5	*GDF5*
27	A0A452E2Z0	Transmembrane protein 65	*TMEM65*
28	A0A5D6WHQ8	Precorrin-4 C(11)-methyltransferase	*cobM*
29	A0A452G6A8	Ceramide synthase 6	*CERS6*
30	A0A452E5L8	Neurofibromin 1	*NF1*
31	A0A452F4W3	Mov10 like RISC complex RNA helicase 1	*MOV10L1*
32	A0A452E392	Protein LSM14 homolog B	*LSM14B*
33	A0A452DVQ0	TOPBP1 interacting checkpoint and replication regulator	*TICRR*
34	A0A5D6WBZ4	S41 family peptidase	*FZ040_05185*
35	A0A452FL24	Flagellum associated containing coiled-coil domains 1	*FLACC1*
36	A0A452G137	Vitamin D-binding protein	*GC*
37	S4SAK0	Pyruvate kinase	*pklr*
38	A0A452F3A5	1-phosphatidylinositol 4,5-bisphosphate phosphodiesterase	*PLCB1*
39	A0A5D6VXX5	Succinate--CoA ligase	*sucC*
40	A0A452ELG6	Melan-A	*MLANA*

## Data Availability

The datasets generated and analyzed during the current study are available in the ProteomeXchange Consortium via the PRIDE repository (https://ftp.pride.ebi.ac.uk/pride/data/archive/2024/03/PXD050828, accessed on 1 June 2024).
